# The Correlation Between Sleep and Coronary Heart Disease: A Review

**DOI:** 10.31083/RCM37252

**Published:** 2025-07-18

**Authors:** Qingbo Shi, Yang Gao, Zhuocheng Shi, Muwei Li

**Affiliations:** ^1^Department of Cardiology, Central China Fuwai Hospital of Zhengzhou University, Fuwai Central China Cardiovascular Hospital, 450000 Zhengzhou, Henan, China; ^2^Department of Cardiology, Zhengzhou University People's Hospital, Henan Provincial People's Hospital, 450000 Zhengzhou, Henan, China; ^3^Central China Subcenter of National Center for Cardiovascular Diseases, Henan Cardiovascular Disease Center, 450000 Zhengzhou, Henan, China

**Keywords:** coronary heart disease, sleep duration, sleep quality, sleep disorders

## Abstract

Coronary heart disease (CHD), which is characterized by the coronary arteries narrowing or becoming obstructed due to atherosclerosis, leads to myocardial ischemia, hypoxia, or necrosis. Owing to an aging population and lifestyle changes, the incidence of CHD and subsequent mortality rates continue to rise, making CHD one of the leading causes of disability and death worldwide. Hypertension, diabetes, hyperlipidemia, smoking, obesity, and genetic factors are considered major risk factors for CHD; however, these factors do not fully explain the complexity and diversity in the etiology of CHD. Sleep, an indispensable part of human physiological processes, is crucial for maintaining physical and mental health. In recent years, the rapid pace of modern life has led to an increasing number of patients experiencing an insufficient amount of sleep, declining sleep quality, and sleep disorders. Therefore, the correlation between sleep and CHD has become a focal point in current research. This review aims to address the relationship between sleep duration, quality, and sleep disorder-related diseases with CHD and emphasizes potential underlying mechanisms and possible clinical implications. Moreover, this review aimed to provide a theoretical basis and clinical guidance for the prevention and treatment of CHD.

## 1. Introduction

Ischemic heart disease, also called coronary heart disease (CHD), is caused by the narrowing or obstructions of coronary arteries as a result of the buildup of atherosclerosis, which can lead to myocardial ischemia and hypoxia, and ultimately myocardial necrosis. Aging and lifestyle changes have led to an increase in CHD which has resulted in increased mortality, and is now a major global health threat [1]. Although significant progress has been made in the diagnosis and treatment of CHD over the past few decades, the global economic burden of ischemic heart disease continues to increase [2]. The etiology and pathogenesis of CHD have long been a focus of research. The development of CHD is a complex process affected by multiple risk factors including aging, smoking, obesity, hypertension, diabetes, dyslipidemia, and genetic predisposition. These risk factors promote the formation and progression of coronary atherosclerosis through mechanisms such as lipid infiltration, endothelial injury, smooth muscle cell proliferation and migration, platelet aggregation, and thrombosis [3]. However, the exact causes and mechanisms of CHD remain unclear and require further investigation. A comprehensive understanding of the etiology and pathogenesis of CHD is crucial for developing more effective preventive measures, accurate diagnostic methods, and treatment strategies.

Sleep is a basic physiological process that is essential to overall health and cardiovascular function [4]. Good sleep not only helps to restore physical strength, promote tissue repair, and cell regeneration, but also enhances immune function, regulates metabolic processes, and maintains the balance of the endocrine system [5, 6]. However, with the fast-paced lifestyle, an increasing number of people are suffering from insufficient sleep, declining sleep quality, and sleep disorders. In China, approximately one-quarter of adolescents experience sleep disorders [7]. Furthermore, with increasing age, the incidence of sleep disorders tends to rise, with more than one-third of elderly individuals reporting sleep disturbances [8]. Research has shown that insufficient sleep, excessive sleep duration, poor sleep quality, and sleep disorder-related diseases all increase the risk of cardiovascular diseases (CVD), including CHD, angina, and myocardial infarction. In contrast, a healthy sleep pattern (such as early bedtime, 7–8 hours of sleep per night, rarely or never suffering from insomnia, no sleep apnea, and not frequently experiencing excessive daytime sleepiness) can significantly reduce the incidence of CHD, CVD, and stroke [9, 10]. In 2022, the American Heart Association updated its “8 Factors for Cardiovascular Health” assessment system, and for the first time, incorporating sleep health in the guidelines [11]. Therefore, there exists a close and complex relationship between sleep and CHD, as well as major adverse cardiovascular events (MACE), which holds significant value for the prevention and treatment of CHD.

**Fig. 1.**
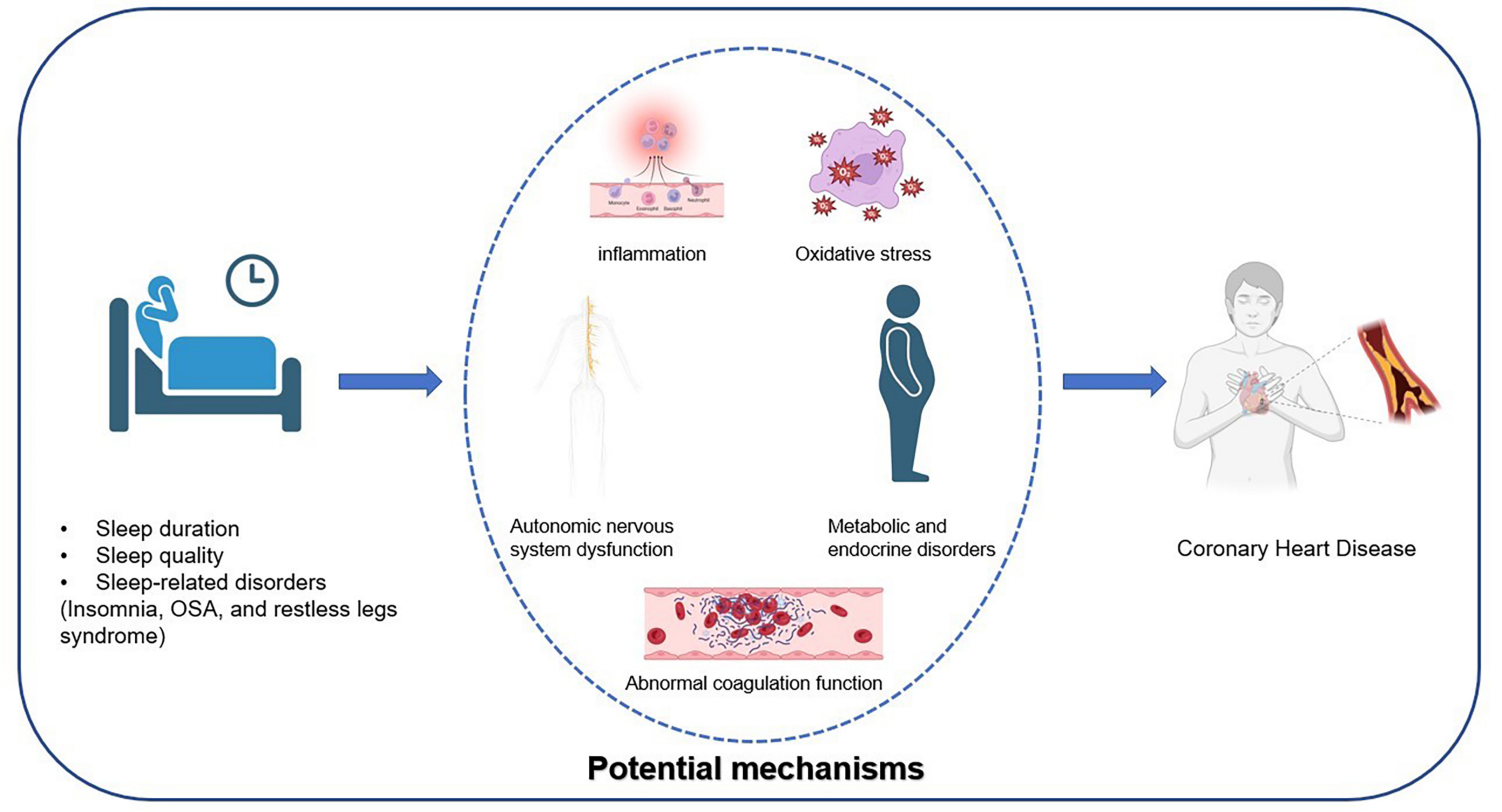
**The Relationship Between Sleep and Coronary Heart Disease and Its Potential Mechanisms**. OSA, obstructive sleep apnea.

This review addresses the relationship between sleep duration, quality, and sleep disorder-related diseases with CHD and emphasizes potential underlying mechanisms and possible clinical implications. The aim is to offer a theoretical foundation and clinical guidance for the prevention and treatment of CHD. The graphical abstract is shown in Fig. 1.

## 2. The Correlation Between Sleep Duration and CHD

The American Heart Association guidelines indicate that the optimal sleep duration for adults is between 7 to 9 hours per night [11]. Another study suggests that the best time to sleep is between 10 and 11 PM [12]. However, with changes in lifestyle, the average sleep duration has gradually decreased, and insufficient sleep has become an increasingly common phenomenon [13]. A survey involving 444,306 participants showed that over one-third of the subjects slept less than 7 hours per night, 23.0% of them slept 6 hours per night, and 11.8% slept less than 5 hours each night [14]. The reduction in sleep duration is closely linked to changes in lifestyle, such as intense work and study schedules, the use of electronic devices before bed, and more active nighttime social activities. Furthermore, sleep disorders related diseases, such as insomnia and obstructive sleep apnea (OSA), are significant contributors to sleep deprivation [15]. The relationship between insufficient sleep and CHD has been a focal point of research, with growing evidence suggesting that inadequate sleep is closely linked to the onset and progression of CHD. However, the relationship between prolonged sleep duration and CHD remains controversial. While some studies suggest that prolonged sleep duration may elevate the risk of CHD, others have reported no significant association between extended sleep and the risk of CHD [16, 17].

Several studies have demonstrated a strong relationship between decreased periods of sleep and the risk for CHD. Ayas *et al*. [18] conducted a decade-long follow-up of 71,617 initially healthy women to examine the association between self-reported sleep duration and the incidence of CHD. After accounting for multiple potential confounders, such as snoring, body mass index, and smoking, the adjusted relative risks for CHD (95% confidence interval [CI]) were 1.45 (1.10–1.92) for those sleeping five hours or less, 1.18 (0.98–1.42) for six hours, and 1.09 (0.91–1.30) for seven hours. The Whitehall II study followed 10,308 healthy adults for 15 years and found that the relative risk of CHD was highest among those with insufficient sleep accompanied by sleep disorders (relative risk [RR]: 1.55, 95% CI: 1.33–1.81) [19]. Research has also shown that individuals with very short sleep durations (≤5 hours) are also closely associated with the occurrence of unstable angina odds ratio [OR]: 3.241, 95% CI: 1.772–5.925) and myocardial infarction (OR: 2.525, 95% CI: 1.113–5.728) [20]. Barger *et al*. [21] conducted a 2.5-year follow-up study on 13,026 patients with acute coronary syndrome (ACS) (within 30 days of onset). Patients who reported <6 hours of sleep per night had a 29% higher risk of major coronary events (MCE; CHD death, myocardial infarction, or urgent revascularization) (adjusted hazard ratio [HR]: 1.29; 95% CI: 1.12–1.49; *p* < 0.001) compared with those with longer periods of sleep. Additionally, for individuals who slept less than 6 hours on weekdays, sleeping an additional 2 hours on weekends was associated with a significant reduction in the incidence of angina, CHD, and other CVD [22]. These studies consistently suggest that insufficient sleep increases the risk of CHD and MACE, making insufficient sleep a potential risk factor for CHD.

Several prospective cohort studies have shown that both short and long periods of sleep are associated with an increased risk of CHD. Wang *et al*. [23] conducted an 18-year follow-up study of 12,268 twin individuals without CVD at baseline (mean age = 70.3 years) to examine the incidence of CVD. In a fully adjusted Cox model, compared to those sleeping 7 to 9 hours per night, the hazard ratios (HRs) for CVD were 1.14 (95% CI: 1.01–1.28) for individuals sleeping fewer than 7 hours and 1.10 (95% CI: 1.00–1.21) for those sleeping 10 or more hours per night. Another large prospective cohort study [24] included 392,164 adults and analyzed the relationship between sleep duration and CHD mortality. The results revealed that, compared to the normal sleep duration of 6 to 8 hours per night, individuals who slept less than 4 hours and more than 8 hours had a 34% (HR: 1.34, 95% CI: 0.87–2.07) and 35% (HR: 1.35, 95% CI: 1.11–1.65) increased risk of death from CHD, respectively. Furthermore, subgroup analyses by gender and age showed that this U-shaped relationship was more pronounced in women and the elderly. Other studies have also indicated that individuals sleeping 7 hours per night have the lowest all-cause, CVD, and other causes of mortality rates [25]. Meta-analyses also indicate a U-shaped relationship between sleep duration and CHD [26, 27, 28]. Yin *et al*. [27] identified a U-shaped relationship between sleep duration and the risks of all-cause mortality, total cardiovascular disease, CHD, and stroke, with the lowest risk observed at around seven hours of sleep per day. Each one-hour decrease in sleep duration was associated with a pooled RR for CHD of 1.07 (95% CI: 1.03–1.12), while each one-hour increase corresponded to a pooled RR of 1.05 (95% CI: 1.00–1.10). Therefore, both short and long sleep durations are associated with CHD and MACE, but the optimal sleep duration remains a subject of ongoing debate.

Although a U-shaped relationship has been widely reported, several studies dispute the relationship of long sleep duration to CHD risk and point to areas for future study. For example, a study involving 20,432 participants aged 20–65 years without a history of CVD found no significant correlation between long sleep duration (≥9 hours) and the incidence of CHD or CVD during a 10–15 year follow-up period [16]. Genetic variants associated with continuous, short (≤6 h) and long (≥9 h) sleep durations were used to examine the causal associations with 12 CVDs among 404,044 UK Biobank participants of White British ancestry. Additional analyses reinforced the detrimental impact of genetically predicted short sleep duration on the risk of 5 out of 12 CVDs, specifically arterial hypertension, pulmonary embolism, coronary artery disease, myocardial infarction, and chronic ischemic heart disease (*p* < 0.001), while also indicating a potential association with atrial fibrillation (*p* < 0.05). In contrast, no significant relationship was observed between genetically predicted long sleep duration and any form of CVD [17]. These findings challenge the view that prolonged sleep duration increases the risk of CHD and CVD, suggesting that the relationship between long sleep duration and CHD, as well as other CVDs, requires further investigation and confirmation.

## 3. The Correlation Between Sleep Quality and CHD

Sleep quality includes subjective and objective measures such as sleep duration, efficiency, continuity, and depth. In 1989, Buysse *et al*. [29] developed the Pittsburgh Sleep Quality Index (PSQI), which assesses sleep quality across seven components: subjective sleep quality, sleep latency, sleep duration, habitual sleep efficiency, sleep disturbances, use of sleeping medications, and daytime dysfunction. The PSQI reflects an individual’s sleep quality over the past month and is currently one of the most commonly used tools for evaluating sleep quality. In 2022, Nelson *et al*. [30] also conducted a conceptual analysis of sleep quality and identified four key attributes: sleep efficiency, sleep latency, sleep duration, and awakenings after sleep onset. Good sleep quality is essential for an individual’s physical and mental health, quality of life, and overall well-being. However, sleep quality has been declining in modern society. Various factors, including physical conditions (such as bodily illnesses), psychological factors (such as stress, anxiety, and depression), environmental factors (such as noise, light, and temperature), and the use of certain medications, can all negatively impact sleep quality. Research has clearly indicated a significant association between sleep quality and both CVD and CHD [16, 31, 32].

Twig *et al*. [32] conducted a follow-up study involving 26,023 males (mean age: 30.9 ± 5.6 years) over an average period of 6.4 ± 4.1 years to evaluate the association between baseline sleep quality and the incidence of diabetes and CHD. The results revealed that poor sleep quality plays a role in the onset of both conditions, with its impact increasing over time. Zhang *et al*. [33] also conducted a survey on 27,935 participants (11,177 males and 16,758 women) from rural areas in Henan Province to explore the independent and interactive relationships between nighttime sleep duration, sleep quality, and CHD. The results indicated that both poor sleep quality and insufficient sleep duration were associated with an increased prevalence of CHD. Additionally, individuals with both insufficient sleep duration and poor sleep quality had the highest proportion of CHD. Another study followed 9570 participants without CHD for 8 years, during which 411 participants (4.29%) developed CHD [34]. After adjusting for conventional CHD risk factors and sleep duration, the relative risks of CHD were significantly higher in the moderate sleep quality group (HR: 1.393; 95% CI: 1.005–1.931) and the poor sleep quality group (HR: 1.913; 95% CI: 1.206–3.035) compared to the good sleep quality group. Poor sleep quality may represent a novel and modifiable risk factor for CHD, independent of traditional cardiovascular risk factors, even when sleep duration remains within the normal range.

Poor sleep quality is a risk factor for disease progression and MACE in patients with coronary heart disease. Andrechuk *et al*. [35] assessed the relationship between sleep quality during hospitalization and the occurrence of MACE including cardiovascular death, recurrent cardiovascular ischemic events, and stroke in patients with acute myocardial infarction (AMI). The results showed that 12.4% of patients experienced MACE, and this was independently associated with poor sleep quality. Yang *et al*. [36] studied the relationship between sleep quality and the severity of coronary artery lesions as well as prognosis in young patients with ACS. The findings indicated that persistent poor sleep quality is a contributing factor to the development of complex coronary artery lesions. Moreover, prolonged poor sleep quality (PSQI >5) was strongly linked to a higher risk of MACE, with a HR of 4.266 (95% CI: 2.274–8.001). In diabetic populations, a healthy sleep pattern is significantly associated with a reduced risk of CHD and CVD mortality [37]. Some researchers argue that sleep quality has a more significant impact on CHD than sleep duration [38, 39]. Therefore, improving sleep quality is not only an effective way to enhance individual health but also one of the key strategies for preventing CHD. Future research should further explore the long-term benefits of improved sleep quality on cardiovascular health and develop new interventions to enhance public sleep quality.

## 4. The Common Sleep Disorders and Their Association With CHD

Sleep disorders are common in adults and are frequently linked to adverse outcomes, such as diminished quality of life and heightened risk for mortality. Among the most prevalent types are insomnia, OSA, and restless legs syndrome (RLS) [40]. The connection between sleep disorders and CHD has been a longstanding focus of research in the medical and public health domains.

### 4.1 The Correlation Between Insomnia and CHD

Insomnia is the most common type of sleep disorder. The American Academy of Sleep Medicine defines insomnia as a condition characterized by sufficient opportunities for sleep but with significant issues in the initiation, consolidation, duration, or quality of sleep, accompanied by daytime functional impairments [41]. The diagnosis of chronic insomnia can be made when these symptoms occur at least three times per week and persist for more than three months. Daytime functional impairments include fatigue or general discomfort, difficulties with concentration or memory, irritability or emotional instability, daytime sleepiness, or any other form of impaired social or occupational functioning. Studies have found that the prevalence of insomnia ranges from 15% to 24%, with an increasing trend over time [42, 43, 44]. Short-term insomnia may lead to various emotional problems, such as excessive daytime sleepiness, mood swings, irritability, and heightened concerns about sleep quality. Chronic insomnia, on the other hand, can result in severe physical health issues, including cognitive dysfunction, endocrine disturbances, and CVD [45]. Insomnia, as a common sleep disorder, has been widely confirmed to be closely associated with an increased risk of developing CHD.

Multiple studies have shown that insomnia not only increases the prevalence of CHD but is also closely associated with ACS and MACE [46, 47, 48, 49, 50]. More than one-third of patients with ACS report moderate to severe insomnia symptoms during their hospitalization [48]. Laugsand *et al*. [49] conducted a follow-up study of 52,610 healthy participants over a period of 11.4 years to assess the impact of insomnia symptoms on the risk of AMI. After adjusting for confounding factors, the risk of AMI was found to increase by 27% to 45% due to various insomnia symptoms. The symptom most strongly associated with myocardial infarction was difficulty falling asleep. Furthermore, when multiple insomnia symptoms were considered together, the relationship between insomnia and myocardial infarction exhibited a dose-dependent pattern. Frøjd *et al*. [51] also conducted a follow-up study of 1068 patients who had experienced a myocardial infarction or undergone coronary artery revascularization, with an average follow-up period of 4.2 years. The results showed that, compared to patients without insomnia, those with insomnia had a 62% increased relative risk of experiencing MACE after adjusting for age and sex (RR: 1.62; 95% CI: 1.24–2.11). Even after adjusting for risk factors, cardiovascular comorbidities, and symptoms of anxiety and depression, the association between insomnia and MACE remained significant (RR: 1.41; 95% CI: 1.05–1.89).

Several meta-analyses have further confirmed the association between insomnia and an increased risk of CHD and MACE. The results of these meta-analyses indicate that insomnia is significantly associated with an increased incidence of myocardial infarction (RR: 1.69, 95% CI: 1.41–2.0). Sleep disturbances related to sleep initiation and maintenance are also associated with a higher incidence of myocardial infarction (RR: 1.13; 95% CI: 1.04–1.23) [52]. Another meta-analysis, which included 13 prospective studies and followed 122,501 participants without CVD for 3 to 20 years, found that insomnia was associated with a 45% increased risk of CVD or death (RR: 1.45; 95% CI: 1.29–1.62) [53]. Another meta-analysis, which included 17 cohort studies involving 311,260 individuals without baseline CVD, found that the relative risk of CVD-related mortality was 33% higher in individuals with insomnia (95% CI: 1.13–1.57) [54]. These findings highlight the potential harm of insomnia to cardiovascular health and underscore the importance of recognizing and managing insomnia in clinical practice to mitigate its contribution to the risk of CVD.

### 4.2 The Correlation Between OSA and CHD

OSA also known as obstructive sleep hypopnea syndrome, is another common sleep disorder-related condition. It is characterized by recurrent partial and complete upper airway obstruction, leading to intermittent hypoxemia, autonomic nervous system dysfunction, and sleep fragmentation [55, 56]. The symptoms of OSA include frequent apneas, snoring, nocturnal awakenings, morning headaches, daytime sleepiness, and difficulty concentrating, all of which severely affect the patient’s sleep quality and daily life. Studies suggest that approximately half of the global population is affected by OSA [57]. As the population of overweight and obese individuals continues to grow, the prevalence of OSA is also steadily increasing. The prevalence of OSA is even higher among patients with CHD, stroke, heart failure, and arrhythmias, reaching 65%, 75%, 55%, and 50%, respectively [58]. Unfortunately, the diagnosis of OSA remains suboptimal. Among individuals with clinically significant OSA identified in population studies, as many as 86% to 95% of patients have not been diagnosed with the condition [59]. In cardiovascular medical practice, the recognition and treatment of OSA remain insufficient.

Study has shown that moderate to severe OSA is associated with increased volume of total atherosclerosis in patients with CHD [60]. The association remained significant even after adjusting for cardiovascular risk factors such as BMI, diabetes and high blood pressure. Mooe *et al*. [61] found that the severity of hypoxemia during sleep is a major determinant of ST-segment depression on the electrocardiogram, and that OSA patients are more likely to experience AMI during the night. Hao *et al*. [62] recruited 1927 patients with ACS and conducted a follow-up for an average of 2.9 years to assess the impact of OSA on the prognosis of ACS patients. The results showed that OSA was independently associated with the occurrence of MACE in ACS patients. In those with a history of myocardial infarction, OSA increased the risk of MACE by 1.74 times (adjusted HR: 1.74; 95% CI: 1.04–2.90). OSA is also associated with an increased risk of MACE in CHD patients following percutaneous coronary intervention. In patients with ST-segment elevation myocardial infarction who also have OSA, the survival rate without cardiovascular events over the subsequent 18 months is significantly lower [63]. Studies have also shown that OSA is more likely to lead to coronary artery calcification [64], plaque instability [65], and vulnerable plaques [66]. Data from the previous meta-analysis showed that there is also a strong association between OSA and cardiometabolic markers such as triglyceride-glucose index (TyG), lipid accumulation product (LAP) and lipid accumulation product (AIP) [67, 68]. Therefore, long-term OSA can affect the vasculature, heart, and brain, contributing to the development of CHD, heart failure, stroke, diabetes, and even an increased risk of mortality. The inclusion of sleep assessment in cardiovascular risk stratification is helpful for the early identification and intervention of patients with CHD and the prevention of adverse cardiovascular events [69].

Continuous positive airway pressure (CPAP) is the primary treatment for OSA and can significantly improve patients’ sleep quality, daytime sleepiness, and overall quality of life. Studies have confirmed that CPAP not only improves sleep symptoms in moderate to severe OSA patients but can also lower blood pressure. Additionally, it can reduce troponin and brain natriuretic peptide (BNP) levels, offering some improvement in myocardial injury [70]. A study published in *The Lancet* followed OSA patients for 10.1 years and found that, among male patients, severe OSA significantly increased the risk of both fatal and non-fatal cardiovascular events, but that CPAP treatment was shown to reduce this risk [71]. However, one study showed that in CHD patients with non-somnolent OSA, routine CPAP treatment does not significantly improve long-term cardiovascular outcomes [72]. Meta-analyses suggest that CPAP use in CHD patients with OSA may help prevent subsequent cardiovascular events. However, this finding has only been confirmed in observational studies and has not been validated in randomized controlled trials [73]. Therefore, there is still some controversy regarding whether CPAP treatment can reduce the occurrence of MACE [74]. However, for CHD patients, screening for OSA and providing appropriate treatment are necessary [58].

### 4.3 The Correlation Between RLS and CHD

RLS is a common neuro-sensory-motor disorder characterized by an uncontrollable urge to move the legs and uncomfortable sensations in the legs, primarily occurring at night and during periods of rest. It significantly impacts sleep and quality of life and is one of the common sleep disorder-related conditions [75]. A meta-analysis on the epidemiology of RLS estimated that the prevalence of RLS in the general population ranges from 5% to 8%. Most patients experience mild RLS symptoms, and the prevalence of RLS gradually increases with age [76]. Studies have shown that RLS often coexists with conditions that are associated with an increased risk of CHD, such as obesity, hypercholesterolemia, and diabetes [77, 78]. It has also been shown that the prevalence of RLS is related to coronary artery disease and coronary artery disease severity [79]. It is speculated that RLS may be related to vascular endothelial dysfunction in CHD. Almuwaqqat *et al*. [80] conducted a 5-year follow-up study of 3266 CHD patients undergoing coronary angiography. After adjusting for demographic and clinical risk factors, the results revealed that moderate to severe RLS patients had a significantly higher risk of major adverse events (cardiovascular death or myocardial infarction) compared to those without RLS (HR: 1.33; 95% CI: 1.01–1.76). This association was particularly more pronounced in males. Therefore, moderate to severe RLS may be an independent risk factor for cardiovascular adverse outcomes. However, one study showed that primary RLS is not associated with the onset of CVD or coronary artery disease [81]. Therefore, the relationship between RLS and CHD requires further investigation.

## 5. Potential Mechanisms of Poor Sleep Leading to CHD

The impact of poor sleep on heart health is widely recognized in the scientific community; however, the specific mechanisms by which poor sleep leads to CHD remain unclear. A summary of previous studies suggests that poor sleep may promote the development of atherosclerosis and CHD through mechanisms such as inflammation, oxidative stress, autonomic nervous system dysfunction, metabolic and endocrine disturbances, and coagulation abnormalities.

### 5.1 Inflammation

Inflammation has long been considered a key factor in the development of atherosclerosis [82, 83]. Inflammatory mediators, such as tumor necrosis factor-alpha (TNF-α), interleukin-1β (IL-1β), interleukin-6 (IL-6), interleukin-17 (IL-17), and C-reactive protein (CRP), play important roles in chronic inflammatory responses. These mediators can promote lipid accumulation, thereby driving the development and progression of atherosclerosis and CHD [84]. The specific mechanisms by which poor sleep leads to CHD and MACE remain unclear, but inflammation may be one of the underlying mechanisms [85]. An animal study has shown that prolonged sleep deprivation for four consecutive days can lead to neutrophil aggregation and an inflammatory storm in mice, resulting in multi-organ dysfunction [86]. In healthy adults, compared to adequate sleep, even a single night of partial sleep deprivation (with sleep limited to 4 hours) leads to a significant increase in the levels of IL-6 and TNF-α produced by monocytes the following day [87]. Partial sleep deprivation not only promotes the activation of lymphocytes and the production of inflammatory mediators such as IL-1β, IL-6, and IL-17, but even after two nights of restorative sleep, these inflammatory mediators remain elevated, accompanied by increased heart rate and serum CRP levels [88]. Parthasarathy *et al*. [89] also found in a prospective study that participants with chronic persistent insomnia had a significantly higher cardiovascular and respiratory mortality rate, which was closely associated with a marked increase in the level of inflammation. In addition, the levels of inflammatory mediators are significantly elevated in OSA patients, and these inflammatory markers increase the risk of atherosclerosis and the development of CHD [90, 91, 92]. Wen *et al*. [93] also showed that, compared to the OSA-only group, patients with both OSA and CHD had significantly elevated levels of CRP, TNF-α, IL-6, and interferon-γ. Long-term periods of poor sleep may lead to persistent changes in the immune system and a chronic inflammatory state, increasing the risk of developing CHD and MACE. Therefore, sleep-targeted interventions may become a new strategy for suppressing inflammation and could have a significant impact on reducing the risk of inflammation-related diseases.

### 5.2 Oxidative Stress

The dynamic balance between reactive oxygen species (ROS) and antioxidants plays a crucial role in maintaining normal cellular function. Oxidative stress induced by excessive ROS has become one of the primary mechanisms driving the development of atherosclerosis [94]. Oxidative stress can promote the formation and progression of atherosclerosis through mechanisms such as inflammation, endothelial dysfunction, and the proliferation and migration of endothelial cells and smooth muscle cells [95]. Poor sleep is closely associated with oxidative stress, and therefore, oxidative stress may be another important mechanism by which poor sleep contributes to the development of CHD [96]. Vaccaro *et al.* [97] used experiments in fruit flies and mice to demonstrate that sleep deprivation leads to the accumulation of ROS and increases oxidative stress levels in the body. The mortality caused by severe sleep restriction may be due to oxidative stress. A randomized crossover design study [98] also showed that six weeks of sleep restriction impaired the ability of endothelial cells to clear ROS, increased oxidative stress levels in endothelial cells, and led to endothelial dysfunction. This may, over time, increase the risk of developing CVD. Short-term sleep restriction has also been associated with elevated levels of myeloperoxidase, an enzyme involved in the formation of oxidants. Myeloperoxidase can modify low-density lipoprotein cholesterol into oxidized low-density lipoprotein cholesterol, exacerbating endothelial damage and lipid accumulation, thus promoting the progression of atherosclerosis [99, 100]. Nighttime intermittent hypoxia in OSA patients is closely associated with increased oxidative stress, elevated inflammatory cytokines, imbalance in nitric oxide production, and endothelial injury. CPAP has been shown to significantly improve oxidative stress, inflammation, and endothelial dysfunction caused by OSA [101]. Therefore, poor sleep may promote the development of atherosclerosis and CHD through oxidative stress. Good sleep not only alleviates oxidative stress but may also have a protective effect on cardiovascular health.

### 5.3 Autonomic Nervous System Dysfunction

The autonomic nervous system plays a crucial role in maintaining cardiovascular homeostasis by regulating heart rate, blood pressure, vascular tone, and cardiac contractility to ensure normal cardiovascular function. Dysregulation of the autonomic nervous system, particularly excessive activation of the sympathetic nervous system, is closely associated with various CVDs such as CHD, arrhythmias, and hypertension [102, 103]. Since it may be a potential mechanism by which sleep problems contribute to CHD and CVD, autonomic nervous system function has been widely studied [104]. Most research data indicate that insufficient sleep leads to excessive activation of the sympathetic nervous system [105, 106]. Compared to patients with adequate sleep, those with insufficient sleep or insomnia show significantly higher concentrations of norepinephrine in both their blood and urine [107, 108]. Sympathetic nervous system overactivation caused by poor sleep leads to an increased heart rate and reduced heart rate variability [109]. An increased heart rate shortens ventricular diastolic time and myocardial blood flow perfusion time, increasing the risk of atherosclerosis, thrombosis, and myocardial ischemia. An epidemiological study has reported that hypertension, a common risk factor for CHD, is closely linked to sympathetic nervous system overactivation caused by insufficient sleep [110, 111]. Blood pressure fluctuations, hypoxemia, and hypercapnia caused by OSA can also activate the sympathetic nervous system through pressure receptors, as well as central and peripheral chemoreceptors [112, 113, 114]. Sympathetic activity increased significantly with increasing severity of OSA [115]. Autonomic dysfunction caused by insufficient sleep may also lead to endothelial dysfunction [116, 117], inflammation [118], and an increased risk of CHD and CVD. Therefore, interventions targeting autonomic dysfunction caused by sleep disturbances may become an effective strategy to reduce the risk of CHD and improve patient outcomes.

### 5.4 Metabolic and Endocrine Disorders

The metabolic syndrome is a pathological condition characterized by abdominal obesity, insulin resistance, hypertension, and hyperlipidemia. Each component of metabolic syndrome contributes to an increased risk of CHD [119]. Studies have shown a close association between insufficient sleep and obesity, which may be related to increased hunger and appetite in sleep-deprived individuals, along with a reduction in daily physical activity and energy expenditure [120, 121, 122]. Insomnia patients with shorter sleep duration have a significantly higher risk of developing diabetes [123]. Insomnia can also lead to an increase of nearly 23% in fasting blood glucose levels and a nearly 48% increase in fasting insulin levels in diabetic patients [124]. This suggests that insomnia not only increases the risk of developing diabetes, but is also closely associated with poor blood glucose control and insulin resistance in diabetic patients. Wang *et al.* [125] also suggested that sleep patterns (such as sleep duration, sleep type, insomnia, snoring, and daytime sleepiness) interact with glucose tolerance in relation to CVD. Poor sleep patterns may increase the risk of diabetes, thereby contributing to a higher prevalence of CVD. Liang *et al*. [126], using genetic data, predicted a moderate association between short sleep duration and metabolic syndrome, as well as several of its core components, including central obesity, dyslipidemia, hypertriglyceridemia, and hyperglycemia. However, long sleep duration was not found to be associated with metabolic syndrome or any of its components. Liu *et al*. [127] also showed that genetically predicted insomnia is consistently associated with higher body mass index, triglycerides, and lower high-density lipoprotein cholesterol levels, with each of these factors potentially playing a mediating role in the causal pathway between insomnia and various cardiovascular disease outcomes. There is also evidence suggesting that insomnia patients, especially those with objectively short sleep duration, exhibit significantly increased hypothalamic-pituitary-adrenal (HPA) axis activity, accompanied by elevated secretion of stress hormones such as adrenal corticosteroids and cortisol [128, 129, 130, 131]. Chronic activation of the HPA axis and endocrine dysfunction also increase the risk of developing metabolic syndrome and CHD.

### 5.5 Abnormal Coagulation Function

Under normal physiological conditions, the body’s coagulation and fibrinolytic systems maintain a dynamic balance to prevent excessive bleeding and thrombus formation. Coagulation dysfunction, including increased activity of coagulation factors, enhanced platelet function, or impaired fibrinolytic system activity, are key factors in the development of CHD and MACE [132, 133]. Platelets play a crucial role in atherosclerosis and acute thrombotic events [134, 135]. One night of sleep deprivation can promote the release of extracellular vesicles into the bloodstream, inducing platelet activation and increasing the risk of thrombosis [136]. Other studies [137, 138] suggest that OSA not only promotes platelet activation but also impairs fibrinolytic system function, leading to a hypercoagulable state. This may be related to intermittent hypoxia, increased sympathetic nervous activity, systemic inflammation, and endothelial dysfunction induced by OSA. A hypercoagulable state and impaired fibrinolytic system function can lead to thrombotic events, which is one of the potential mechanisms linking OSA to adverse cardiovascular and cerebrovascular events. In healthy individuals, sleep disruption significantly increases the levels of soluble tissue factor and von Willebrand factor in the blood, suggesting that sleep disruption is associated with elevated biomarkers of thrombotic risk in the cardiovascular system [139]. Additionally, plasma levels of von Willebrand factor are significantly higher in individuals with a nightly sleep duration of either less than 7 hours or more than 7 hours compared to those with a sleep duration of 7 hours per night [140]. Therefore, coagulation dysfunction is one of the important mechanisms through which poor sleep contributes to the development of CHD and MACE.

## 6. Conclusion

Sleep duration, sleep quality, and sleep-related disorders are intricately linked to the development of CHD and the incidence of MACE. Poor sleep may contribute to the development of CHD and MACE through several pathways, including inflammation, oxidative stress, autonomic dysfunction, metabolic and endocrine disorders, and coagulation abnormalities. Therefore, incorporating sleep assessment into cardiovascular risk stratification and early identification and intervention strategies are essential for the prevention of CHD and MACE. However, the precise mechanisms through which poor sleep leads to CHD remain unclear, and maintaining healthy sleep patterns continues to be a challenge. Further research is necessary to define these mechanisms and to develop better strategies to promote improved sleep duration and quality into clinical practice to decrease the incidence of CHD and MACE.
